# Multiple pathways of lipid dysregulation in amyotrophic lateral sclerosis

**DOI:** 10.1093/braincomms/fcac340

**Published:** 2022-12-26

**Authors:** Katherine Phan, Ying He, Surabhi Bhatia, Russell Pickford, Gordon McDonald, Srestha Mazumder, Hannah C Timmins, John R Hodges, Olivier Piguet, Nicolas Dzamko, Glenda M Halliday, Matthew C Kiernan, Woojin Scott Kim

**Affiliations:** The University of Sydney, Brain and Mind Centre, Sydney, NSW, Australia; The University of Sydney, School of Medical Sciences, Sydney, NSW, Australia; The University of Sydney, Brain and Mind Centre, Sydney, NSW, Australia; The University of Sydney, School of Medical Sciences, Sydney, NSW, Australia; The University of Sydney, Brain and Mind Centre, Sydney, NSW, Australia; The University of Sydney, School of Medical Sciences, Sydney, NSW, Australia; Bioanalytical Mass Spectrometry Facility, University of New South Wales, Sydney, NSW, Australia; The University of Sydney, Sydney Informatics Hub, Sydney, NSW, Australia; The University of Sydney, Brain and Mind Centre, Sydney, NSW, Australia; The University of Sydney, Brain and Mind Centre, Sydney, NSW, Australia; The University of Sydney, Brain and Mind Centre, Sydney, NSW, Australia; The University of Sydney, Brain and Mind Centre, Sydney, NSW, Australia; The University of Sydney, School of Psychology, Sydney, NSW, Australia; The University of Sydney, Brain and Mind Centre, Sydney, NSW, Australia; The University of Sydney, School of Medical Sciences, Sydney, NSW, Australia; The University of Sydney, Brain and Mind Centre, Sydney, NSW, Australia; The University of Sydney, School of Medical Sciences, Sydney, NSW, Australia; The University of Sydney, Brain and Mind Centre, Sydney, NSW, Australia; Institute of Clinical Neurosciences, Royal Prince Alfred Hospital, Sydney, NSW, Australia; The University of Sydney, Brain and Mind Centre, Sydney, NSW, Australia; The University of Sydney, School of Medical Sciences, Sydney, NSW, Australia

**Keywords:** amyotrophic lateral sclerosis, lipidomics, biomarkers

## Abstract

Amyotrophic lateral sclerosis is a rapidly progressing neurodegenerative disease characterized by the degeneration of motor neurons and loss of various muscular functions. Dyslipidaemia is prevalent in amyotrophic lateral sclerosis with aberrant changes mainly in cholesterol ester and triglyceride. Despite this, little is known about global lipid changes in amyotrophic lateral sclerosis or in relation to disease progression. The present study incorporated a longitudinal lipidomic analysis of amyotrophic lateral sclerosis serum with a comparison with healthy controls using advanced liquid chromatography-mass spectrometry. The results established that diglyceride, the precursor of triglyceride, was enriched the most, while ceramide was depleted the most in amyotrophic lateral sclerosis compared with controls, with the diglyceride species (18:1/18:1) correlating significantly to neurofilament light levels. The prenol lipid CoQ_8_ was also decreased in amyotrophic lateral sclerosis and correlated to neurofilament light levels. Most interestingly, the phospholipid phosphatidylethanolamine and its three derivatives decreased with disease progression, in contrast to changes with normal ageing. Unsaturated lipids that are prone to lipid peroxidation were elevated with disease progression with increases in the formation of toxic lipid products. Furthermore, *in vitro* studies revealed that phosphatidylethanolamine synthesis modulated *TARDBP* expression in SH-SY5Y neuronal cells. Finally, diglyceride, cholesterol ester and ceramide were identified as potential lipid biomarkers for amyotrophic lateral sclerosis diagnosis and monitoring disease progression. In summary, this study represents a longitudinal lipidomics analysis of amyotrophic lateral sclerosis serum and has provided new insights into multiple pathways of lipid dysregulation in amyotrophic lateral sclerosis.

## Teaser

Phan *et al*. report a longitudinal lipidomics analysis of amyotrophic lateral sclerosis serum and have provided new insights into multiple pathways of lipid dysregulation in amyotrophic lateral sclerosis.

## Graphical Abstract

**Graphical abstract:**
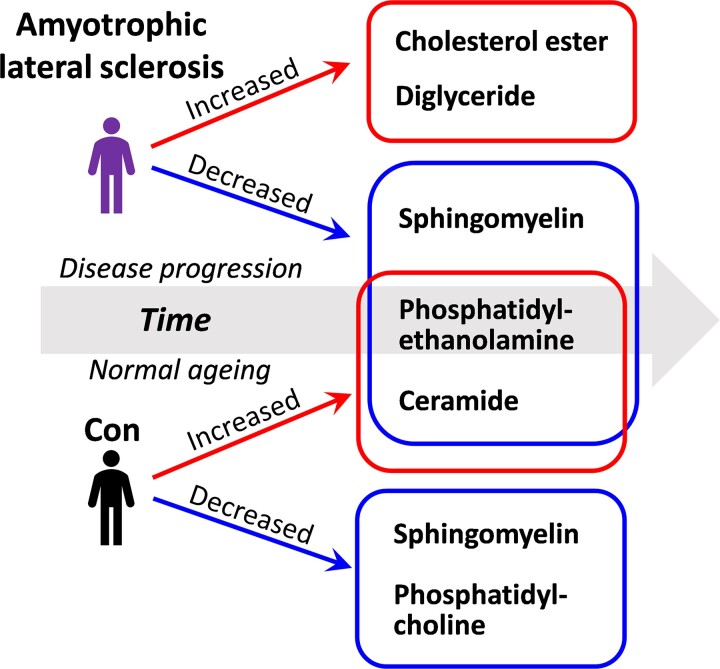

## Introduction

Amyotrophic lateral sclerosis (ALS) is a neurodegenerative disease characterized by progressive degeneration of motor neurons that results in muscle atrophy, dysarthria, gradual paralysis and death usually within 2–5 years from diagnosis.^1,2^ ALS overlaps with another neurodegenerative disease, frontotemporal dementia (FTD), with TDP-43 as the predominant pathology in both diseases.^3^ The overwhelming majority, ∼90%, of ALS cases are sporadic. In familial cases, mutations in over 20 genes have been associated with ALS, including TAR DNA binding protein (*TARDBP*) that encode TDP-43.^4^ Currently, there is no cure for ALS and the FDA-approved drug Riluzole extends survival, albeit only by ∼3 months.^5^ Although the exact mechanism by which Riluzole improves survival is unclear, it appears to reduce glutamate release, attenuate persistent sodium and inward calcium currents,^6,7^ whilst at the same time leading to an improvement in functional capacity of mitochondria.^8^

Mitochondrial dysfunction is a predominant feature of ALS and has been associated with multiple pathogenic pathways in ALS, with a number of ALS candidate genes implicated in mitochondrial dysfunction, e.g. TDP-43, which forms aggregates in mitochondria and causes disruption to mtDNA transcription, ATP production and calcium homeostasis.^9^ The function of mitochondria is utmost essential in cell survival and metabolism, which includes ATP production via oxidative phosphorylation, calcium homeostasis and lipid biosynthesis. The role of mitochondria in neurons is of great importance by the fact that neurons consume high levels of ATP. In calcium homeostasis, mitochondria play a crucial role in regulating calcium concentrations that impact neurotransmitter release.

In terms of lipid biosynthesis, mitochondria are a major producer of the lipids phosphatidylethanolamine (PE)^10^ and coenzyme Q_10_ (CoQ_10_),^11^ which are important in the mitochondrial respiratory chain. PE is generated by four independent pathways in eukaryotic cells, including the *PISD* pathway in the mitochondria.^12^ Lipid dysregulation is recognized as a common feature of ALS, although most studies have focused mainly on cholesterol and triglycerides (TGs).^13,14^ Presently, very little is known about PE in the context of ALS. In contrast, the importance of CoQ_10_ in ALS has been well documented and CoQ_10_ has been trialled as a therapeutic supplement to improve mitochondrial function and survival.^15^

In respect to cholesterol and TG, studies produced inconsistent results with contradictory changes in the level of total cholesterol and TG.^13,16,17^ The one consistent result, however, has been the strong association between elevated serum cholesterol and TG levels with prolonged survival of ALS patients.^18-20^ The increase in cholesterol ester (ChE) has been shown to be associated with ALS risk in a retrospective metabolic study.^21^ ChE is also known to mediate oxidative stress-induced death in ALS motor neurons.^22^

Early studies were based on lipid measurements using indirect enzymatic/colorimetric assays. These assays are, however, limited to measuring only a few lipid classes, e.g. cholesterol and TG that are present in moderate-to-high abundance in serum. Furthermore, they cannot measure individual lipid species, of which there are thousands in the serum. Recently, analysis of lipids using mass spectrometry-based lipidomics has come to the fore in the field of neurodegenerative diseases, including ALS.^23-25^ It has enabled the measurement of all lipid classes and lipid species present in the serum with greater accuracy and specificity. Importantly, it has allowed the detection of small, yet significant, changes in lipids that are critical to disease processes. Recent lipidomics analyses of FTD serum have revealed that lipid dysregulation underlies the FTD pathophysiology linked to neurodegeneration.^26,27^

The present study incorporated a longitudinal analysis of lipidomics in the serum of ALS patients. Our primary aim was to identify lipid pathways that were altered in ALS, to understand lipid dysregulation that may contribute to ALS pathogenesis.^28^ Our secondary aim was to identify lipids for the purpose of developing biomarkers for ALS diagnosis and for monitoring disease progression.

## Materials and methods

### Amyotrophic lateral sclerosis patient cohort

Individuals diagnosed with sporadic ALS^29,30^ (*n* = 28; Supplementary Table 1) were recruited from the forefront multidisciplinary ALS clinic at the University of Sydney Brain and Mind Centre. Results were compared with those of healthy control volunteers^31^ (*n* = 22; Supplementary Table 1) without neurological or psychiatric disorders or cognitive impairment. Blood samples were collected at two-time points (i.e. T_1_ and T_2_) for each individual. The mean number of months between the two visits was 12 months for ALS and 23 months for controls. The study was approved by the University of New South Wales (approval number: HC12573) and the University of Sydney (approval numbers: 2012/160, 2014/539 and 2017/928) human research ethics committees. All methods were carried out in accordance with the relevant guidelines and regulations. Blood samples were obtained following written informed consent from the participant and/or primary career as legal representatives. All participants underwent a neurological examination, a comprehensive cognitive assessment and a structural brain MRI and met the current consensus diagnostic criteria for ALS^32^ or no neurological disease. Blood samples (9 mL) were collected in tubes (BD Vacutainer SST II Advance Tube #367958), and serum was prepared by centrifugation at 3500 rpm for 10 min at 4°C, which was then aliquoted and stored at −80°C until use.

### Neurofilament assays

Frozen serum samples were sent to Quanterix (Billerica, MA, USA) and the concentration of neurofilament light (NFL) was measured using the Simoa HD-1 analyzer and software. Samples were randomized, blinded and measured in duplicates using NFL standards. The concentration of phosphorylated neurofilament heavy (pNFH) was measured using a pNFH ELISA kit (RD191138300R, Assay Matrix) and CLARIOstar plate reader (BMG Labtech).

### Lipid extraction and liquid chromatography/mass spectrometry

Lipid extraction and liquid chromatography/mass spectrometry were carried out as per our previously published methods.^26,27,33^ Relative abundance of lipids was obtained from peak areas normalized to internal standards and data were analysed using LipidSearch software 4.1.16.

### Thin-layer chromatography

Thin-layer chromatography was conducted on ALS serum to determine lipid concentrations using the Sulfo-Phospho-Vanillin method, as previously described.^34^

### Calcium assay

Calcium assay was carried out on ALS and control serum using a calcium assay kit (Abcam, ab102505) and by following the manufacturer’s protocol as previously described.^26^

### Malondialdehyde assay

Malondialdehyde (MDA) was carried out on ALS and control serum using a MDA assay kit (Abcam, ab118970) and by following the manufacturer’s protocol as previously described.^26^

### Cell studies

SH-SY5Y neuronal cells were cultured in 12-well plates in Dulbecco’s modified Eagle’s medium containing 10% foetal calf serum, 1% glutamax, 0.5% glucose, 100 IU/mL penicillin and 100 μg/mL streptomycin at 37°C in humidified air containing 5% CO_2_. Cells were cultured in six-well plates and transfected with a vector containing PISD cDNA or TARDBP cDNA or vector-only control using Lipofectamine 2000 (Thermo Fisher Scientific) following the manufacturer’s protocol. After 48 h, the cells were harvested and total RNA was prepared for gene expression studies.

### RNA extraction and quantitative PCR

Total RNA was isolated from cells using TRIzol reagent (Invitrogen) following the manufacturer’s protocol. All procedures were carried out using RNase-free reagents and consumables. The 4 µg of RNA was reverse transcribed into cDNA using Moloney-murine leukaemia virus reverse transcriptase and random primers (Promega, Madison, WI, USA) in a 20 μL reaction volume. Gene expression was measured by quantitative polymerase chain reaction (qPCR) using the BioRad CFX Connect (BioRad, Australia) and the fluorescent dye SYBR Green (BioRad), following the manufacturer’s protocol. Briefly, each reaction (20 μL) contained 1× mastermix, 5 pmoles of primers and 1 μL of cDNA template. Amplification was carried out with 40 cycles at 94°C for 15 s and at 60°C for 1 min. Gene expression was normalized to the geometric mean of three housekeeping genes, *GAPDH*, *β-ACTIN* and *PPIA*. A no-template control was included for each PCR amplification assay.

### Statistical analyses

Statistical analyses of the lipidomics data set were conducted in R version 4.0.3, using the *lipidr* package^35^ for differential expression and discriminant analysis. Lipid expression intensities were normalized with probabilistic quotient normalization^36^ and log_2_ transformed before being analysed for differential expression^37^ in each of the three comparisons. The first compared cases with ALS to healthy controls and the last two compared T_1_ to T_2_ within either of the ALS or the control cohorts, to look at the progression over time. Subsequent lipid set enrichment analyses^38^ were performed for each class of lipids to determine whether the class overall was differentially expressed. TGs were additionally analysed in lipid sets according to chain length and unsaturation and a linear model of the form [log_2_(FC) = *β*_0_ + *β*_1_ × unsaturation + *β*_2_ × chain length], so as to extract the statistically significant trend in this lipid class. Multivariate analyses (general linear model) with age and sex as covariates were used to determine differences in lipids and proteins using SPSS Statistics 26 (IBM, Chicago, IL, USA) and a significance set at *P* < 0.05. Association analyses were performed using Pearson’s correlation and statistical significance set at *P* < 0.05. *In vitro* cell studies were conducted in *n* = 6 replicates and significance was determined using the Student’s *t*-test with *P* < 0.05 considered significant. Individual data plots were generated using GraphPad Prism 9.

## Results

### Lipidomics analysis of amyotrophic lateral sclerosis serum

**Figure 1:**
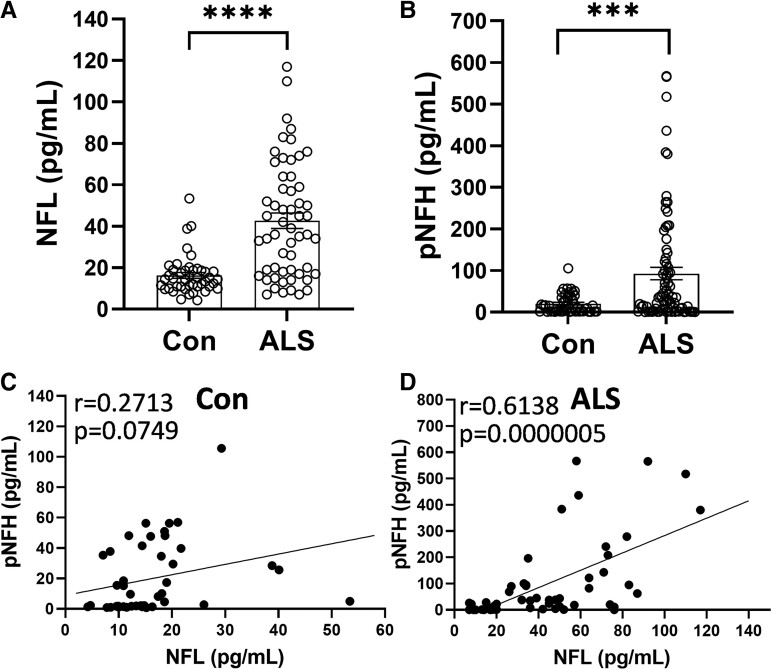
**Evidence of neurodegeneration in the ALS cohort.** (**A**) NFL *t*-test, ****P < 0.00005, *t* = 6.039 and (**B**) pNFH *t*-test, ***P < 0.0005, *t* = 3.698 chains were elevated in ALS (*n* = 56) compared with controls (*n* = 44). Pearson’s correlation between NFL and pNFH in controls (**C**) and in ALS (**D**).

Given that lipid dysregulation is increasingly recognized as a key feature in a number of neurodegenerative diseases, a comprehensive lipidomics analysis was undertaken of ALS serum with the aim of understanding lipid dysregulation in ALS and identifying potential lipid biomarkers for ALS. Firstly, we measured NFL and pNFH chains to verify the manifestation of neurodegeneration in our ALS cohort. Both NFL and pNFH levels were significantly elevated in ALS compared with controls (Fig. 1A and B) and the two proteins correlated strongly with one another in the ALS cohort (Fig. 1C and D).

**Figure 2:**
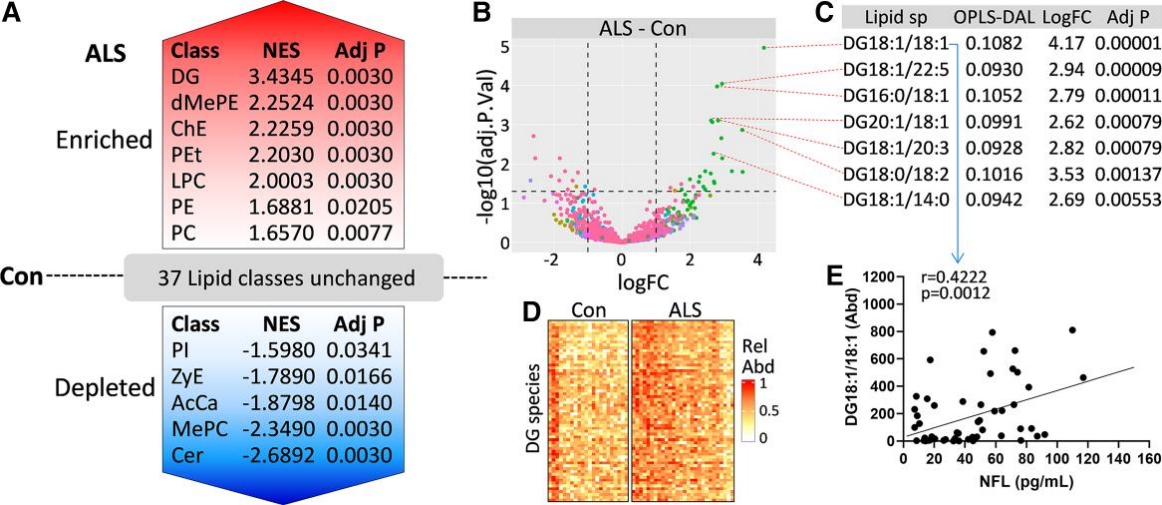
**Lipidomics analysis of ALS serum.** (**A**) LSEA of lipid classes altered in ALS (*n* = 56) serum compared with controls (*n* = 44) was performed using the *lipidr* package for differential expression and discriminant analysis. Lipid expression intensities were normalized with probabilistic quotient normalization and log_2_ transformed before being analysed for differential expression and significance set at adjusted *P* < 0.05. The significant lipids were ranked by NES and significance. A volcano plot shows altered lipid species (**B**) with DG species being the most significantly altered species (**C**). (**D**) A heatmap showing DG species in ALS compared with controls. (**E**) DG (18:1/18:1) species correlated significantly with NFL levels in ALS.

We then extracted total lipids from the same serum samples and the abundance of lipids was analysed using liquid chromatography-mass spectrometry and the LipidSearch software. Statistical analyses on the lipid data set were carried out using the R statistics package *lipidr*, covarying for age and sex. To detect preferential regulation of lipid classes in ALS serum compared with controls, we applied the lipid set enrichment analysis (LSEA) method to the data set. Enriched lipids were ranked by normalized enrichment score (NES) and significance. We found that seven lipid classes [diglyceride (DG), dimethylphosphatidylethanolamine (dMePE), ChE, phosphatidylethanol (PEt), lysophosphatidylcholine (LPC), PE, phosphatidylcholine (PC)] were enriched in ALS compared with controls with DG having the highest NES (Fig. 2A). The five lipid classes [ceramide (Cer), methylphosphatidylcholine (MePC), acylcarnitine (AcCa), ZyE, phytosphingosine (PI)] were depleted in ALS compared with controls with Cer having the lowest NES (Fig. 2A). These lipids belong to different functional groups with AcCa linked closely to mitochondrial function. The 37 other lipid classes were not altered in ALS compared with controls (Fig. 2A). A volcano plot shows that the most significantly altered lipid species was DG (18:1/18:1; Fig. 2B and C). A heatmap of DG species shows an overall increase in the relative abundance of DG in ALS compared with controls (Fig. 2D). Also, DG (18:1/18:1) correlated significantly with NFL levels in ALS (Fig. 2E). DG (18:1/18:1) could therefore be considered as a candidate biomarker for ALS diagnosis.

### Detection of coenzyme Q lipids

**Figure 3:**
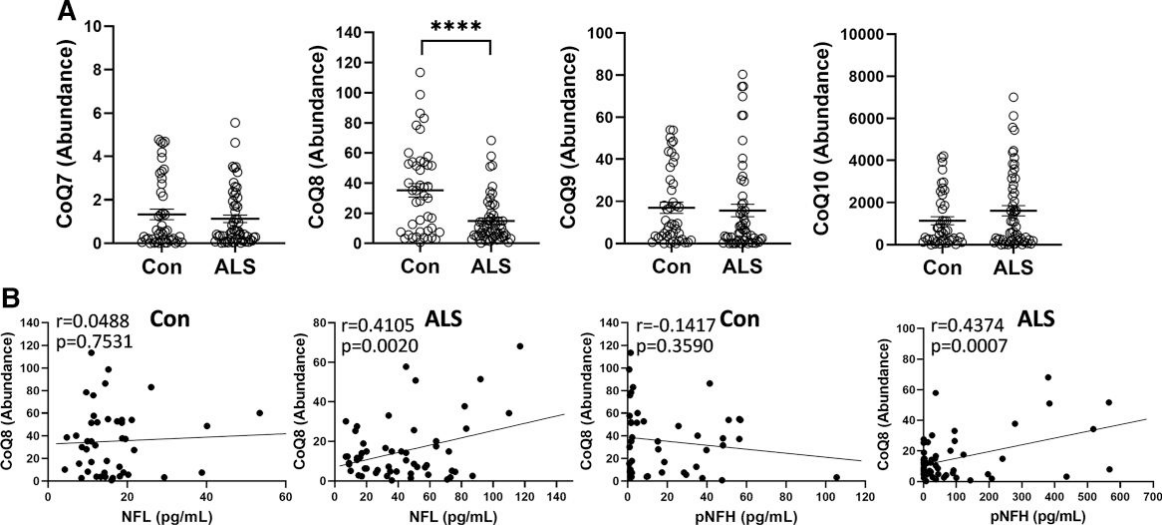
**Measurement of coenzyme Q lipids in ALS serum.** (**A**) A comparison of coenzyme Q lipids CoQ_7_, CoQ_8_, CoQ_9_ and CoQ_10_ in ALS (*n* = 56) serum compared with controls (*n* = 44), *t*-test, ****P < 0.0001, *t* = 4.504. (**B**) Pearson’s correlation between CoQ_8_ and NFL and pNFH in controls and in ALS.

Another group of lipids that was detected by the lipidomics analysis was CoQ (also known as prenol lipids). These lipids are components of the electron transport chain that produces ATP in the mitochondria. We detected CoQ with 7, 8, 9 and 10 isoprene units, i.e. CoQ_7_, CoQ_8_, CoQ_9_ and CoQ_10_, respectively, with CoQ_10_ being the most abundant (Fig. 3A). Only CoQ_8_ was altered, i.e. decreased, in ALS serum compared with controls (Fig. 3A). CoQ_8_ also correlated significantly to NFL and pNFH in ALS (Fig. 3B). Other CoQ levels were not correlated to either NFL or pNFH (data not shown). CoQ_8_ could also be considered as a candidate biomarker for ALS diagnosis.

### Changes in lipids with disease progression

**Figure 4:**
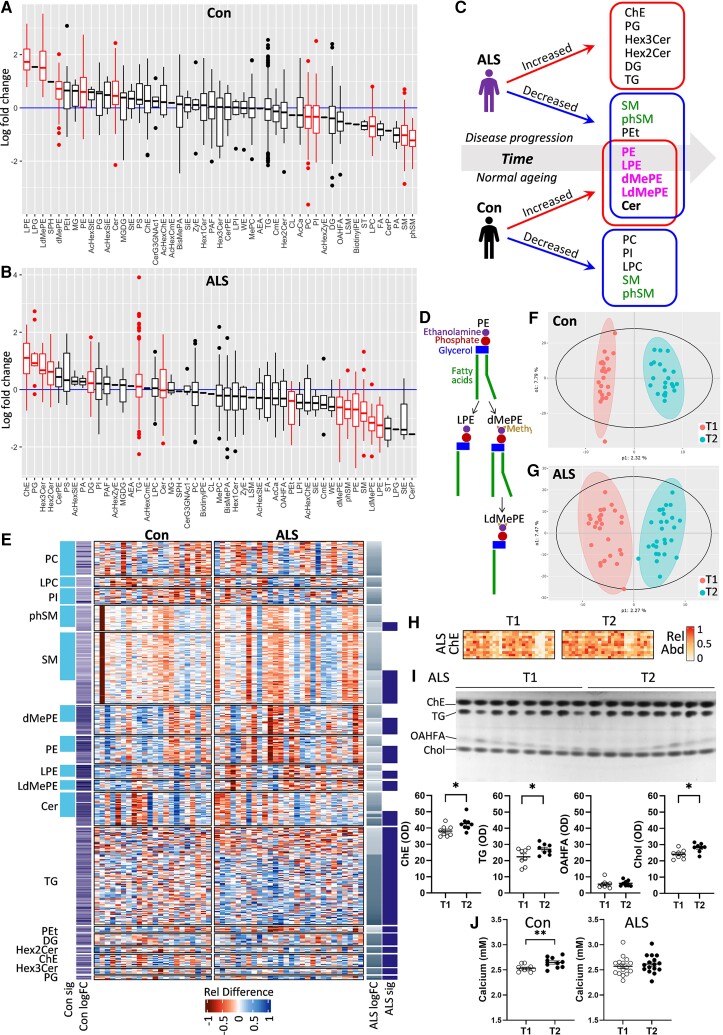
**A longitudinal lipidomics assessment of ALS serum.** LSEA of lipids altered in T_2_ compared with T_1_ in controls (**A**) and in ALS (**B**) was performed using the *lipidr* package for differential expression and discriminant analysis. Lipid expression intensities were normalized with probabilistic quotient normalization and log_2_ transformed before being analysed for differential expression and significance set at adjusted *P* < 0.05; significantly altered lipids are in red. (**C**) A summary of changes in lipids with time. Red boxes contain significantly increased lipids and blue boxes contain significantly decreased lipids. Green text indicates lipids decreased in both ALS and controls. Pink text indicates PE or PE derivatives. (**D**) Derivation of LPE, dMePE and LdMePE from PE. (**E**) A heatmap showing the relative difference between T_2_ and T_1_ for each species for all of the lipid classes that were significantly altered with time. (**F**) OPLS-DA of lipidome in T_1_ and T_2_ in controls (**F**) and in ALS (**G**). (**H**) A heatmap of ChE species in T_2_ compared with T_1_ in ALS. (**I**) Validation of mass spectrometry results using thin layer chromatography of ALS T_1_ (*n* = 8) and T_2_ (*n* = 8), *t*-test, *P < 0.05, *t* = 2.383 (ChE), *t* = 2.151 (TG), *t* = 2.913 (Chol). (**J**) Calcium levels of control T_1_ (*n* = 10) and T_2_ (*n* = 10), *t*-test, **P < 0.01, *t* = 2.931 and ALS T_1_ (*n* = 16) and T_2_ (*n* = 16).

Little is known about the global lipid changes in ALS with respect to disease progression. Here, we analysed lipids in ASL and control serum at two-time points (i.e. T_1_ and T_2_). We used the LSEA enrichment analysis to detect preferential regulation of lipids in T_2_ compared with T_1_ and lipids were ranked by their fold changes with time. We found that 10 lipid classes were significantly altered with time in controls (Fig. 4A), whereas 14 were significantly altered with time in ALS (Fig. 4B). Most interestingly, five [PE, lysophosphatidylethanolamine (LPE), dMePE, lysodimethylphosphatidylethanolamine (LdMePE) and Cer] of the eight lipids that increased with time in controls decreased with time in ALS (Fig. 4C). These were PE and PE derivatives (LPE, dMePE and LdMePE; Fig. 4D). PE is predominantly produced in the mitochondria. Sphingomyelin (SM) and phSM were the only lipids that decreased with time in both ALS and controls (Fig. 4C). In contrast, no same lipids were increased with time in ALS and controls (Fig. 4C). A heatmap shows the relative difference between T_2_ and T_1_ for each species for all of the lipid classes that were significantly altered with time (Fig. 4E), e.g. the abundance of TG species, overall, increased with time in ALS (Fig. 4E). Effects of time on the serum lipidome were demonstrated with the supervised orthogonal partial least squares discriminant analysis (OPLS-DA) method and a clear separation between the two-time points was evident for both the control (Fig. 4F) and ALS (Fig. 4G) cohorts. ChE was the most enriched lipid with time in ALS serum (Fig. 4B) and this was further illustrated by a heatmap of ChE species that shows an overall increase in the relative abundance of ChE in T_2_ compared with T_1_ (Fig. 4H). This result was validated using an alternative method of measuring lipids—thin layer chromatography—which showed a significant increase in ChE, along with validation of other lipids (Fig. 4I). Finally, we measured calcium levels and showed that they are increased with time in controls, but not in ALS (Fig. 4J).

To identify and formulate a lipid signature for ALS for disease diagnosis and disease progression, the three LSEA analyses (Con versus ALS, Con T_1_ versus T_2_, ALS T_1_ versus T_2_) were examined together. Of all the lipids present in the serum, only DG and ChE were enriched in ALS compared with controls and further enriched with disease progression, whereas Cer was depleted in ALS compared with controls and further depleted with disease progression. Therefore, DG, ChE and Cer and/or a combination of these three could be used for developing biomarkers for monitoring the disease progression of ALS.

### Analysis of lipid unsaturation

**Figure 5:**
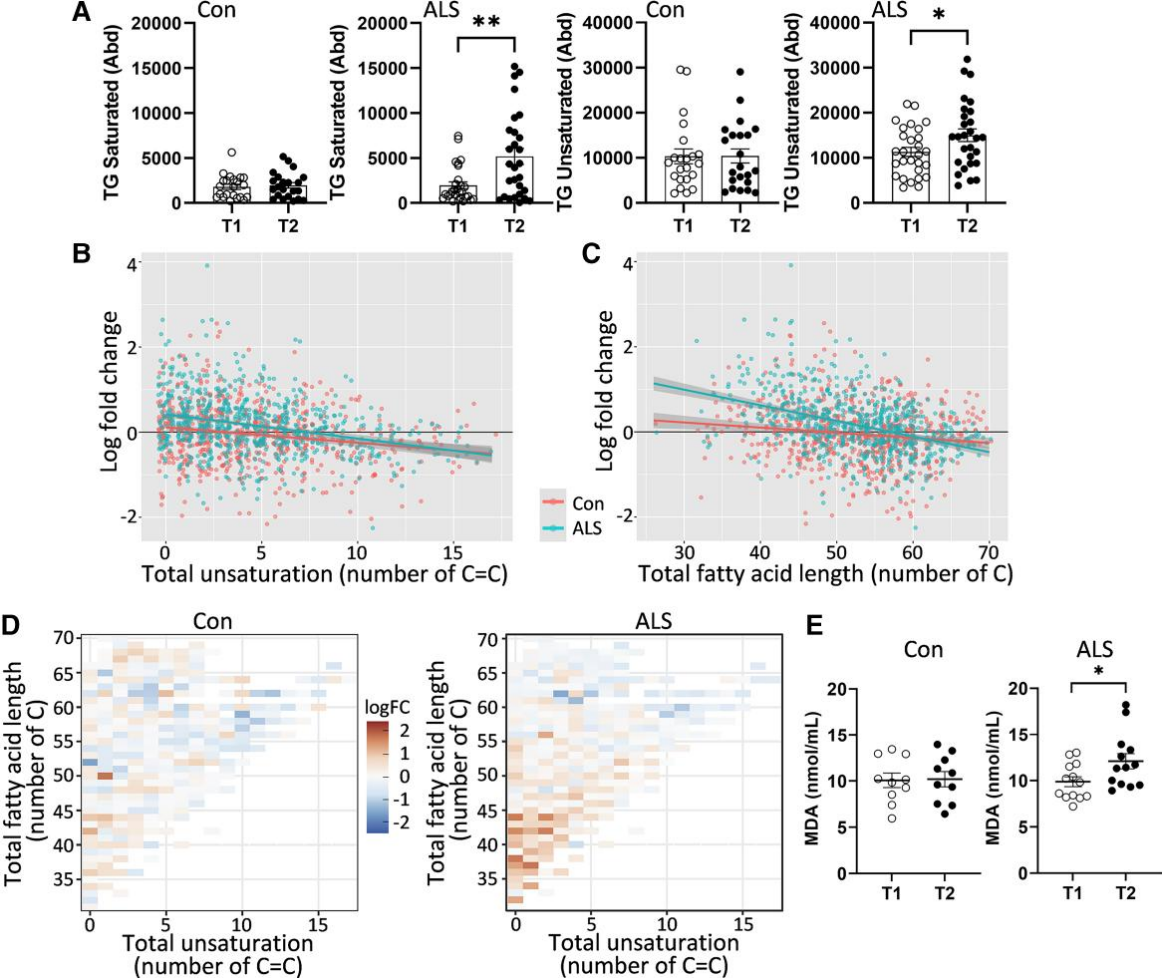
**Analysis of lipid unsaturation.** (**A**) Abundance of saturated *t*-test, *P < 0.05 *t* = 2.118 and unsaturated *t*-test, **P < 0.005, *t* = 3.288 TG in ALS (*n* = 28) and controls (*n* = 22). (**B**) A fold-change plot of individual TG species in terms of total unsaturation. (**C**) A fold-change plot of individual TG species in terms of total fatty acid length. (**D**) Heat maps show that changes were more pronounced in TG species with fewer C=C and shorter chain length in ALS. (**E**) Verification of increased lipid peroxidation product, MDA, with time in ALS (*n* = 13) *t*-test, *P < 0.05, *t* = 2.248 and control (*n* = 10).

Apart from changes to lipid abundance, changes to lipid chemistry also impact on cell pathophysiology. We were particularly interested in the changes to lipid unsaturation with disease progression. Lipids become unsaturated when the hydrogen atoms are replaced by carbon–carbon double bonds (C=C) in the fatty acid chains. Importantly, unsaturated lipids are susceptible to lipid peroxidation and the greater the number of C=C the greater the susceptibility to lipid peroxidation. Lipid peroxidation results in the formation of toxic lipid products, i.e. lipid aldehydes, that cause oxidative stress and neurodegeneration.^39^ We analysed the changes in TG unsaturation with time in ALS serum and controls. TG has three fatty acid chains and is the most abundant lipid in the serum with the highest number of carbon atoms in the fatty acid chains and therefore most relevant to lipid peroxidation. We found that both saturated and unsaturated TG levels were elevated with time in ALS, whereas both unaltered with time in controls (Fig. 5A). This was further demonstrated by a plot of individual TG species that shows, overall, a greater fold-change in ALS compared with controls (Fig. 5B). The overall change in terms of chain length was also greater in ALS compared with controls (Fig. 5C). These changes were more pronounced in TG species with fewer C=C and shorter chain lengths (Fig. 5D).

To investigate the effect of the increased unsaturation in ALS on lipid peroxidation, we then assessed the level of MDA, a major lipid aldehyde, in the same ALS samples at two time points. MDA is highly reactive and toxic to cells.^39^ We found that MDA levels were significantly increased with time (Fig. 5E). These results indicate that lipid peroxidation increases with disease progression in ALS.

### Effect of changes to PE synthesis on *TARDBP* expression

**Figure 6:**
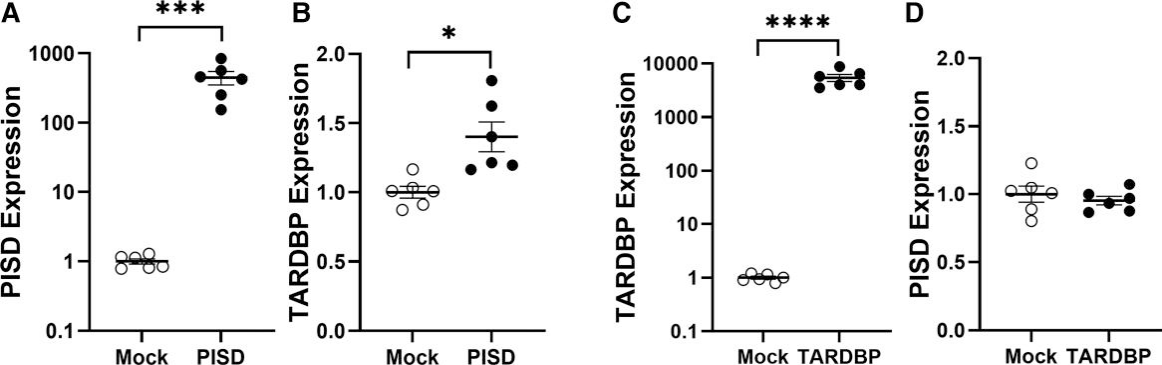
**Effect of changes to PE synthesis on TARDBP expression.** (**A**) SH-SY5Y neuronal cells (*n* = 6) were transfected with PISD cDNA *t*-test, ***P < 0.005, *t* = 4.513 and (**B**) its impact on TARDBP mRNA expression (*n* = 6) *t*-test, **P < 0.01, *t* = 3.483 was measured by qPCR. (**C**) In a separate experiment, SH-SY5Y neuronal cells (*n* = 6) were transfected with TARDBP cDNA *t*-test, ****P < 0.0001, *t* = 6.680 and (**D**) its impact on PISD mRNA expression (*n* = 6) was measured by qPCR.

Since TDP-43 (encoded by the *TARDBP* gene) is the major pathology in ALS with ∼95% ALS cases bearing this phenotype,^40^ we were interested in the effect of changes to PE synthesis on *TARDBP* expression. PE is predominantly synthesized in the mitochondria by the key synthesis gene *PISD*. We altered the expression of *PISD* in SH-SY5Y neuronal cells and measured the effect on *TARDBP* expression and found that overexpression of *PISD* (Fig. 6A) upregulated *TARDBP* transcription (Fig. 6B). In a separate experiment, we altered the expression of *TARDBP* (Fig. 6C) and found no changes to *PISD* transcription (Fig. 6D), indicating that changes in *TARDBP* expression is downstream of PE synthesis.

## Discussion

Increasing evidence shows that lipids are intrinsically linked to neurodegenerative processes with recent advances in mass spectrometry-based lipidomics having substantially improved our understanding of the importance of lipid dysregulation in neurodegenerative diseases. Comprehensive lipidomics analysis of blood serum from ALS patients established that DG, dMePE, ChE, PEt, LPC, PE and PC were increased, while Cer, MePC, AcCa, ZyE and PI were decreased in ALS compared with controls. DG was increased the most with the highest enrichment score and significance. Of all the DG species, DG (18:1/18:1) had the highest OPLS-DA loading score and significance, correlated significantly with NFL and therefore is a strong candidate for ALS biomarker development. Consistent with our results, an earlier lipidomics analysis of plasma showed that the DG class was significantly enriched in ALS compared with controls.^25^ We also detected a group of prenol lipids and found that CoQ_8_ levels were significantly decreased in ALS compared with controls. CoQ_8_ levels were also correlated to NFL levels and thus CoQ_8_ could also be considered as a biomarker for ALS.

We were also interested in understanding the lipid dysregulation associated with disease progression and analysed ALS and control serum in longitudinal samples. We observed that a greater number of lipid classes (i.e. 14) were altered with time in ALS compared with 10 in controls. The six lipid classes were increased with time in ALS, including the glycerolipids DG and TG, which is consistent with other lipidomics analysis of ALS plasma.^25^ We also observed that ChE levels increased with time in ALS. Similarly, ChE (18:1), ChE (18:2) and ChE (22:5) were shown to be significant markers of disease progression of ALS.^24^ Most interestingly, five lipid classes (PE, LPE, dMePE, LdMePE and Cer) that increased with time in controls decreased with time in ALS. In other words, the levels of these lipids were altered conversely with time in ALS compared with controls. LPE, dMePE and LdMePE are all derived from PE. In a mouse model, decreases in PE resulted in a greater availability of DG and increased formation of TG.^41^ Consistent with this, we observed increases in DG and TG with decreases in PE in ALS. These observations suggest that PE is particularly associated with disease progression of ALS. However, virtually nothing is known about PE in the context of ALS.

PE is a major membrane phospholipid that is particularly enriched in the brain, nerves and spinal cord, making up ∼45% of total phospholipid. It is synthesized in the mitochondria via the phosphatidylserine decarboxylase pathway by the *PISD* gene and is highly enriched in the organelle.^42^ It is also synthesized via a pathway steps located in the endoplasmic reticulum. PE has a cone shape because of its relatively small head group (see Fig. 4D) and exerts a lateral pressure that maintains membrane curvature and it is thought that PE regulates mitochondrial structure and morphology.^43^ The importance of PE in mitochondria is demonstrated by the fact that deletion of *PISD* in mice causes extensive mitochondrial fragmentation and lethality in embryonic mice.^44^ We also provided *in vitro* evidence that changes in *PISD* alter *TARDBP* expression in SH-SY5Y neuronal cells. The role of PE is also related to modulating Ca^2+^ ion levels in the function of the ATPase enzyme. Dysregulation of Ca^2+^ homeostasis in the mitochondria is evident at disease onset and progression of ALS that results in loss of mitochondrial membrane potential and subsequent dysfunction.^45^ Furthermore, as a major constituent of membranes, PE mediates membrane movement that allows successful cell growth and division,^46^ which would be important in neurogenesis. A large number of cytosolic proteins bind non-covalently to PE. These so-called PE-binding proteins are highly conserved in evolution and are involved in a number of processes, including neuronal development and the regulation of several signalling pathways.

A number of studies have shown that PE also plays a critical role in the oxidative phosphorylation of the electron transport chain that is relevant to ALS pathogenesis. In one study, knockdown of *PISD* in PSB-2 cells impaired the activities of electron transport chain complex and ATP production.^43^ In another study, PE was shown to be associated with the ubiquinol:cytochrome c reductase complex^47^ and that inclusion of PE in proteoliposomes reconstituted with the electron transport chain complex enhanced oxidative phosphorylation.^48^ Furthermore, PE was shown to modulate NADH:ubiquinone oxidoreductase complex activity in bovine mitochondria.^49^ These and other studies indicate that PE is essential in the regulation of mitochondrial function and structure, although the precise mechanisms by which PE regulates these processes are unclear.

LPE was also decreased with disease progression in ALS. It is derived from PE by the enzymatic removal of one of the two fatty acids (see Fig. 4D). Unlike PE, LPE is a minor constituent of cell membranes and has a different role to PE. It partakes in cell signalling and enzymatic activation. As a lysophospholipid, LPE typically binds to protein targets, such as receptors, kinases and phosphatases, activating specific cellular responses in a broad range of biological processes. Emerging evidence suggests that LPE acts like a neurotrophic factor that promotes neuronal differentiation and migration and that suppresses apoptosis via the MAPK signal cascade.^50^

Another group of lipids that partake in the mitochondrial respiratory chain is CoQ molecules. In our lipidomics analysis, we detected CoQ_7_, CoQ_8_, CoQ_9_ and CoQ_10_ but only CoQ_8_ levels were reduced in ALS compared with controls. Moreover, only CoQ_8_ levels were correlated to NFL levels in ALS. CoQ_8_ levels were, however, minor compared with CoQ_10_, which is regarded as the most important CoQ since it plays a pivotal role in the production of cellular energy in the mitochondria. Much research focus has been on the role of CoQ_10_ in ALS pathogenesis and on CoQ_10_ as a therapeutic supplement. In mouse studies, supplementation of CoQ_10_ reduced the decline in mitochondrial function associated with ageing.^15^ Supplementation of CoQ_10_ in a mouse model of ALS produced promising results of prolonged survival.^51^ However, a Phase II clinical trial of CoQ_10_ supplementation for 9 months had no significant effect on the disease progression of 185 ALS patients.^52^ In fact, most, if not all, clinical trials of the CoQ_10_ supplementation in patients with other neurodegenerative diseases produced, overall, no positive results. A likely reason for these failures could be due to, as a lipid molecule, the extreme hydrophobic nature of CoQ_10_, which renders it low bioavailability when orally administered. It is likely that only a fraction of CoQ_10_ ingested is absorbed from the digestive tract into the bloodstream.

Apart from CoQ_8_, AcCa and Cer were also decreased in ALS compared with controls. AcCa is a key mitochondrial lipid that transports long-chain fatty acids into the mitochondria, where the fatty acids are oxidized to produce ATP energy.^53^ Some Cer species modulate mitochondrial function and oxidative phosphorylation in the production of ATP.^54,55^ Cardiolipin is another lipid that is closely associated with mitochondria and although we detected this lipid, there was no significant change in ALS compared with controls.

 Another aspect of lipid chemistry that could contribute to ALS pathogenesis is lipid peroxidation caused by increases in fatty acid unsaturation. We analysed the degree of unsaturation in TG, which is the most abundant lipid in the serum with the highest number of carbon atoms in the fatty acid chains. We revealed that unsaturated TG levels were significantly increased with time in ALS. Unsaturated lipids are chemically unstable and are prone to peroxidation (a process of oxidative degradation) that results in the formation of lipid aldehydes that are highly toxic to cells. We showed that the lipid aldehyde, MDA, was increased with time in ALS serum. Lipid peroxidation is particularly relevant to neurodegenerative diseases, because the brain is highly enriched in lipids and has a high oxygen consumption. Lipid aldehydes have been shown to contribute to the pathogenesis of a number of neurodegenerative diseases, including FTD.^39,56,57^ We also observed that increases in unsaturation were more prominent in TG species with shorter fatty acid chains. Interestingly, TG species with short-to-medium fatty acid chains have been shown to be associated with increases in resting energy expenditure^58^ as demonstrated by the hypermetabolism and resultant malnutrition common among ALS patients.^59,60^ Overall, these results suggest increased susceptibility to lipid peroxidation in ALS with disease progression and also support the use of lipid measurements to detect changes in lipid peroxidation in ALS serum.

Finally, in order to formulate a lipid signature for ALS, we examined the three LSEA analyses (Con versus ALS, Con T_1_ versus T_2_, ALS T_1_ versus T_2_). Of all the lipids present in the serum, only DG and ChE were increased in ALS compared with controls and further increased with disease progression and therefore could be considered as biomarkers for ALS diagnosis and for monitoring disease progression. Cer could also be considered as a potential biomarker for ALS as it was decreased in ALS compared with controls and further decreased with disease progression. However, these lipid changes are not entirely specific to ALS but could be associated with other neurodegenerative diseases and therefore further work is required to evaluate lipid changes across multiple neurodegenerative diseases. DG (18:1/18:1) and CoQ_8_ were identified as strong candidates for biomarker development for ALS. In a recent lipidomics study of ALS plasma, TG (68:12) was found to be correlated with NFL.^24^ SM and phSM were decreased with time in both ALS and controls and therefore could be considered as markers for normal ageing. Future work in biomarker development could include synthesizing specific lipid species and carrying out targeted quantification.

In conclusion, the present study utilized mass spectrometry-based lipidomics to identify multiple pathways of lipid dysregulation in ALS. Our findings provide new evidence that supports the concepts that aberrations in cellular structure and function contribute to ALS pathology, via specific effects in individual lipidomic pathways, potentially guiding more precise therapeutic approaches.^61^

## Supplementary Material

fcac340_Supplementary_Data

## Data Availability

Lipidomics raw data were generated at Bioanalytical Mass Spectrometry Facility, University of New South Wales. Derived data supporting the findings of this study are available from the corresponding author, upon reasonable request. Other patient data cannot be made publicly available because the ethical approval and the informed consent from the patients included in this study did not cover placing the data into publicly open repositories.

## Abbreviations

AcCa =: acylcarnitine
ALS =: amyotrophic lateral sclerosis
Cer =: ceramide
ChE =: cholesterol ester
CoQ =: coenzyme Q
DG =: diglyceride
dMePE =: dimethylphosphatidylethanolamine
FTD =: frontotemporal dementia
LdMePE =: lysodimethylphosphatidylethanolamine
LPC =: lysophosphatidylcholine
LPE =: lysophosphatidylethanolamine
MePC =: methylphosphatidylcholine
NFL =: neurofilament light
PC =: phosphatidylcholine
PE =: phosphatidylethanolamine
PEt =: phosphatidylethanol
PG =: phosphatidylglycerol
phSM =: sphingomyelin(phytosphingosine)
PI =: phosphatidylinositol
pNHL =: neurofilament heavy
SM =: sphingomyelin
TARDBP =: TAR DNA binding protein
TG =: triglyceride
ZyE =: zymosterol ester.
