# Supplementary material for: Multiple pathways of lipid dysregulation in amyotrophic lateral sclerosis
Source: Brain Commun. 2022 Dec 26;5(1):fcac340. doi: 10.1093/braincomms/fcac340 (PMC9825811; doi:10.1093/braincomms/fcac340)
Supplement: fcac340_Supplementary_Data [file fcac340_supplementary_data.docx]

**Supplementary Table 1**

Demographics of ALS cases and controls used in the study

| **Case** | **Age** | **Sex** | **Disease** |  | **Case** | **Age** | **Sex** | **State** |
| --- | --- | --- | --- | --- | --- | --- | --- | --- |
| A1 | 69 | Male | ALS |  | C1 | 60 | Male | Control |
| A2 | 52 | Male | ALS |  | C2 | 71 | Male | Control |
| A3 | 64 | Male | ALS |  | C3 | 57 | Female | Control |
| A4 | 66 | Male | ALS |  | C4 | 71 | Female | Control |
| A5 | 69 | Male | ALS |  | C5 | 74 | Male | Control |
| A6 | 65 | Male | ALS |  | C6 | 75 | Female | Control |
| A7 | 76 | Male | ALS |  | C7 | 75 | Female | Control |
| A8 | 41 | Female | ALS |  | C8 | 64 | Female | Control |
| A9 | 49 | Female | ALS |  | C9 | 69 | Male | Control |
| A10 | 57 | Male | ALS |  | C10 | 68 | Male | Control |
| A11 | 56 | Male | ALS |  | C11 | 67 | Male | Control |
| A12 | 61 | Female | ALS |  | C12 | 80 | Female | Control |
| A13 | 54 | Female | ALS |  | C13 | 58 | Male | Control |
| A14 | 36 | Male | ALS |  | C14 | 67 | Female | Control |
| A15 | 59 | Male | ALS |  | C15 | 66 | Female | Control |
| A16 | 70 | Male | ALS |  | C16 | 74 | Female | Control |
| A17 | 43 | Male | ALS |  | C17 | 56 | Male | Control |
| A18 | 65 | Female | ALS |  | C18 | 75 | Male | Control |
| A19 | 54 | Male | ALS |  | C19 | 62 | Female | Control |
| A20 | 51 | Male | ALS |  | C20 | 68 | Female | Control |
| A21 | 53 | Female | ALS |  | C21 | 66 | Female | Control |
| A22 | 66 | Male | ALS |  | C22 | 72 | Female | Control |
| A23 | 63 | Male | ALS |  |  |  |  |  |
| A24 | 53 | Male | ALS |  |  |  |  |  |
| A25 | 70 | Male | ALS |  |  |  |  |  |
| A26 | 64 | Female | ALS |  |  |  |  |  |
| A27 | 35 | Male | ALS |  |  |  |  |  |
| A28 | 72 | Male | ALS |  |  |  |  |  |
